# Supplementary material for: Apolipoprotein ɛ4 Status and Brain Structure 12 Months after Mild Traumatic Injury: Brain Age Prediction Using Brain Morphometry and Diffusion Tensor Imaging
Source: J Clin Med. 2021 Jan 22;10(3):418. doi: 10.3390/jcm10030418 (PMC7865561; doi:10.3390/jcm10030418)
Supplement: Supplementary file 1 [file jcm-10-00418-s001.pdf]

Table S1. Multivariable regression of APOE-ε4 status and DTI (AD and RD).

ROI	Comparison Groups			
	APOE-ε4(−) vs APOE-ε4(+)			
	B	SE	*p*-value	R^2
AD-ATR L	1.318×10^{-5}	0.000	0.07	0.071
AD-ATR R	9.353×10^{-6}	0.000	0.26	0.036
AD-CG L	1.891×10^{-6}	0.000	0.75	0.166
AD-CG R	-4.332×10^{-6}	0.000	0.53	0.086
AD-CING L	8.781×10^{-6}	0.000	0.38	0.129
AD-CING R	-2.388×10^{-6}	0.000	0.82	0.083
AD-CST L	7.013×10^{-6}	0.000	0.20	0.302
AD-CST R	6.293×10^{-6}	0.003	0.28	0.253
AD-FMAJ	2.883×10^{-6}	0.000	0.63	0.490
AD-FMIN	7.434×10^{-7}	0.000	0.93	0.333
AD-IFOF L	-1.940×10^{-6}	0.000	0.73	0.052
AD-IFOF R	-2.99×10^{-6}	0.000	0.60	0.012
AD-ILF L	-6.001×10^{-6}	0.000	0.30	0.040
AD-ILF R	-8.863×10^{-6}	0.000	0.12	0.073
AD-SLF L	1.159×10^{-6}	0.000	0.81	0.082
AD-SLF R	-9.312×10^{-7}	0.000	0.85	0.042
AD-SLFT L	-4.647×10^{-6}	0.000	0.48	0.022
AD-SLFT R	-7.823×10^{-6}	0.000	0.31	0.114
AD-UF L	-5.01×10^{-6}	0.000	0.48	0.365
AD-UF R	-4.739×10^{-9}	0.000	0.99	0.350
AD-CCBody	7.839×10^{-7}	0.000	0.93	0.037
AD-CCGenu	6.207×10^{-6}	0.000	0.60	0.100
AD-CCSplenium	8.223×10^{-6}	0.000	0.45	0.301
AD-ws	1.277×10^{-6}	0.000	0.78	0.078
RD-ATR L	4.389×10^{-6}	0.000	0.47	0.203
RD-ATR R	8.342×10^{-7}	0.000	0.91	0.171
RD-CG L	-8.235×10^{-7}	0.000	0.88	0.174
RD-CG R	-3.003×10^{-7}	0.000	0.95	0.126
RD-CING L	1.626×10^{-5}	0.000	0.04	0.228
RD-CING R	1.229×10^{-5}	0.000	0.14	0.137
RD-CST L	2.695×10^{-6}	0.000	0.56	0.135
RD-CST R	5.471×10^{-6}	0.000	0.28	0.190
RD-FMAJ	-1.175×10^{-7}	0.000	0.81	0.261
RD-FMIN	-4.768×10^{-6}	0.000	0.47	0.249
RD-IFOF L	-2.223×10^{-6}	0.000	0.66	0.196
RD-IFOF R	-6.433×10^{-6}	0.000	0.22	0.193
RD-ILF L	-1.008×10^{-6}	0.000	0.85	0.155
RD-ILF R	-3.674×10^{-6}	0.000	0.48	0.114
RD-SLF L	-1.680×10^{-6}	0.000	0.70	0.127
RD-SLF R	-2.487×10^{-6}	0.000	0.58	0.154
RD-SLFT L	-1.759×10^{-6}	0.000	0.73	0.092
RD-SLFT R	-4.859×10^{-6}	0.000	0.35	0.203
RD-UF L	-3.903×10^{-6}	0.000	0.46	0.197
RD-UF R	-3.989×10^{-6}	0.000	0.47	0.123
RD-CCBody	7.470×10^{-6}	0.000	0.38	0.204
RD-CCGenu	-3.141×10^{-6}	0.000	0.68	0.304
RD-CCSplenium	-5.057×10^{-7}	0.000	0.93	0.164
RD-ws	-2.286×10^{-7}	0.000	0.96	0.159

Adjusted for Age, sex and head coil

Abbreviations: Axial Diffusivity (AD), RAdial diffusivity (RD), Anterior thalamic radiation (ATR), Cingulum (cingulate gyrus, CG), Cingulum (hippocampus, CING), Corticospinal tract (CST), Forceps major (FMAJ), Forceps minor (FMIN), Inferior fronto-occipital fasciculus (IFOF), Inferior longitudinal fasciculus (ILF), Superior longitudinal fasciculus (SLF), Superior longitudinal fasciculus (temporal part, SLFT) , Uncinate fasciculus (UF), Corpus callosum (CC), Whole Skeleton (ws).
